# Constitutions Created

**Published:** 1859-06

**Authors:** 


### CONSTITUTIONS CREATED.

To build up a good constitution, we must take good care of what we have, and add to it, by pretty hard work and moderate thought, until the age of forty-five ; then, there should be less work and more thought.

Bodily labor consolidates the constitution up to forty-five ; then, mental labor preserves it, keeps it good to the verge of fourscore years, if the bodily activities are very moderate. As witness Humboldt, who was a great traveller in early life ; but from fifty to ninety a great student. Many similar instances will occur to intelligent minds. The general idea is of great practical importance. Work hard until forty-five ; think hard after, and all the while, be " temperate in all things." This is to live long.

